# Metabolome and transcriptome analyses reveal quality change in the orange-rooted *Salvia miltiorrhiza* (Danshen) from cultivated field

**DOI:** 10.1186/s13020-019-0265-6

**Published:** 2019-10-02

**Authors:** Zhilai Zhan, Wentao Fang, Xiaohui Ma, Tong Chen, Guanghong Cui, Ying Ma, Liping Kang, Tiegui Nan, Huixin Lin, Jinfu Tang, Yan Zhang, Changjiangsheng Lai, Zhenli Ren, Yanan Wang, Yujun Zhao, Ye Shen, Ling Wang, Wen Zeng, Juan Guo, Luqi Huang

**Affiliations:** 1 State Key Laboratory Breeding Base of Dao-di Herbs, National Resource Center for Chinese Materia Medica, China Academy of Chinese Medical Sciences, Beijing, 100700 China; 2 School of Pharmacy, Anhui University of Chinese Medicine, Hefei, 230012 China; 3 College of Pharmaceutical Science, Yunnan University of Traditional Chinese Medicine, Kunming, 650500 China; 4 Shandong Danhong Pharmaceutical Co., Ltd., Heze, 27400 Shandong China

**Keywords:** *Salvia miltiorrhiza* Bunge (Danshen), Tanshinones, Metabolome profiling, mRNA expression profiling, ER-associated degradation

## Abstract

**Background:**

The dry root and rhizome of *Salvia miltiorrhiza* Bunge, or Danshen, is a well-known, traditional Chinese medicine. Tanshinones are active compounds that accumulate in the periderm, resulting in red-colored roots. However, lines with orange roots have been observed in cultivated fields. Here, we performed metabolome and transcriptome analyses to investigate the changes of orange-rooted Danshen.

**Methods:**

Metabolome analysis was performed by ultra-high-performance liquid chromatography-quadrupole time-of-flight mass spectrometry (UPLC/Q-Tof–MS) to investigate the metabolites variation between orange Danshen and normal Danshen. RNA sequencing and KEGG enrichment analysis were performed to analyzing the differentially expressed genes between orange-rooted and normal Danshen.

**Results:**

In total, 40 lipophilic components were detected in metabolome analysis, and seven compounds were significantly decreased in the orange Danshen, including the most abundant active compounds, tanshinone IIA and tanshinone I in normal Danshen. Systematic analysis of transcriptome profiles revealed that the down-regulated genes related to catalytic dehydrogenation was not detected. However, two genes related to stress resistance, and four genes related to endoplasmic reticulum (ER)-associated degradation of proteins were up-regulated in orange Danshen.

**Conclusions:**

Decreases in the content of dehydrogenated furan ring tanshinones such as tanshinone IIA resulted in phenotypic changes and quality degradation of Danshen. Transcriptome analysis indicated that incorrect folding and ER-associated degradation of corresponding enzymes, which could catalyze C_15_-C_16_ dehydrogenase, might be contributed to the decrease in dehydrogenated furan ring tanshinones, rather than lower expression of the relative genes. This limited dehydrogenation of cryptotanshinone and dihydrotanshinone I into tanshinones IIA and I products, respectively, led to a reduced quality of Danshen in cultivated fields.

## Background

Danshen, the dry root and rhizome of *Salvia miltiorrhiza* Bunge, is one of the most versatile components in traditional Chinese medicine. More than 636 medicinal preparations utilize Danshen as their main constituent, which can be found in the Chinese patent medicine prescription library. Among them, Danshen Tablets and Fufang Danshen Dripping Pills have been widely used in modern Chinese clinical trials and are considered effective in treating cardiovascular and cerebrovascular diseases [1].

**Fig. 1:**
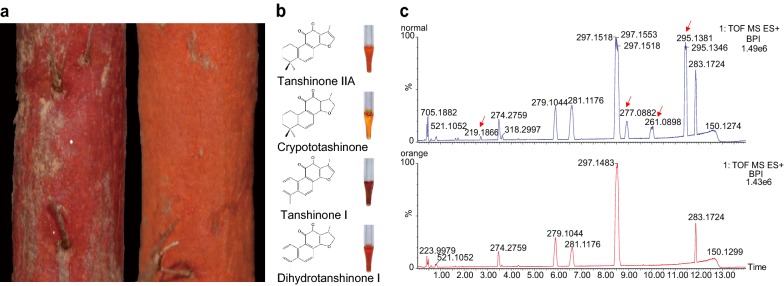
**a** Color phenotypes of normal Danshen (left) and orange Danshen (right). **b** Phenotypes of tanshinone IIA, cryptotashinone, tanshinone I, and dihydrotanshinone I. **c** The chromatogram of total ions found in normal Danshen (top) and orange Danshen (bottom)

The main components of Danshen include hydrophilic phenolic acids and lipophilic diterpenoid tanshinones, which are used to evaluate the quality of medicinal materials [1, 2]. Modern pharmacological studies and chemical investigations suggest that both components contribute to Danshen’s pharmacological and therapeutic effects. Phenolic acid compounds are distributed in the roots and leaves of *S. miltiorrhiza*, while tanshinones mainly accumulate in the roots and rhizomes. More than 40 structurally diverse tanshinones have been isolated and identified since their discovery in the 1930s. Among them, tanshinone IIA, cryptotanshinone, tanshinone I, and dihydrotanshinone I are the main active ingredients [3]. Most tanshinones have colors ranging from orange to red (Fig. 1), which elicit the reddish phenotype of Danshen. These compounds have been extensively investigated for their well-known cardiovascular activities, as well as for their anti-cancer activities in vitro and in vivo [4]. Tanshinone IIA has also been reported to have potential for treating human inflammation [5], as well as anti-adipogenic effects on 3T3-L1 cells and in zebrafish [6].

In China, more than 70,000 tons of Danshen is consumed every year. Before the 1970s, wild Danshen was the main ingredient in traditional Chinese medicine. However, with increased demand, wild resources have greatly decreased. Now, Danshen is cultivated in northern and middle China, providing almost all the material for traditional patent prescriptions and for clinical use in Chinese medicine. Thus, the quality and consistency of cultivated Danshen are essential for medicinal security. Interestingly, some orange-rooted lines have appeared during cultivation, suggesting that variations in tanshinone content or composition may affect the phenotype and quality of Danshen (Fig. 1a). Additionally, this phenomenon is not an isolated case and has been observed in several different cultivation areas, including the Shandong, Henan, and Shanxi Provinces. Therefore, extensive investigation of the orange lines found in cultivated fields is important for further utilization of Danshen.

The genome sequence and transcriptome analysis of Danshen from various development periods [7–9], different tissues [10], and tissue cultures from different induction times were taken [11], which provide a foundation for the biological analysis of this useful medicinal plant. Based on these analyses, the biosynthesis and regulation of tanshinones and phenolic acids have made great strides [12–14]. However, there are still many aspects that need to be studied based on comparative omics data and genome sequencing.

In this study, we utilized ultra-high-performance liquid chromatography-quadrupole time-of-flight mass spectrometry (UPLC/Q-TOF–MS) to analyze the component variations in both normal and orange Danshen to assess their differences in quality. In order to determine a possible explanation for these phenotypic changes, high-throughput RNA sequencing was performed to detect differences in gene expression between the two types of Danshen.

## Materials and methods

### Plant materials

Danshen were collected in July 2015 from the Chinese herbal medicine cultivation base located in Changqing, Shandong Province, China. Three normal Danshen samples and three orange Danshen samples representing three replicates were collected for metabolite profiling and transcriptome analysis during the florescence period in July. At this time, growth conditions transfer from vegetative to reproductive growth and secondary metabolites are accumulated during this period. The root of these Danshen were sampled and frozen with liquid nitrogen and stored at − 80 °C respectively for subsequent metabolome and RNA-sequencing analyses.

### Chemicals and reagents

Reference standards of tanshinone IIA, cryptotanshinone, tanshinone I, dihydrotanshinone I, tanshinone IIB, dihydroisotanshinone I, danshenxinkun B, methylenetanshinquinone, tetrahydrotanshinone I, miltirone, dehydromiltirone, sageone, isocryptotanshinone, tanshindiol A, tanshindiol B, tanshindiol C, 1,2-dihydrotanshinquinone, tanshinol B, sugiol, and methyltanshinonate (Beijing Rongchengxinde Technology Development Co., Ltd, Beijing, China) had purities above 95%.

Acetonitrile and methanol (Merck, Darmstadt, Germany), as well as formic acid (Fisher Scientific, Geel, Belgium), were HPLC grade. Ultra-pure water (18.2 MΩ/cm) was purified using a Barnstead GenPure UV/UF water purification system (Thermo Fisher Scientific, Langenselbold, Germany).

### Sample and standard preparation

Danshen roots used for metabolome analysis were dried by freeze drying (EYELA, Tokyo, Japan) to constant weight, and then pulverized and sieved through a 50 mesh. Powdered samples (1.0 g) were dissolved in 10 mL of 80% methanol and ultrasonically extracted for 30 min at 25 °C and 40 kHz (SCIENT ultrasonic processor, Ningbo, China). After centrifugation at 13,000 rpm for 10 min, supernatants were filtered through a 0.22 μm membrane filter. In order to identify the compounds in *S. miltiorrhiza* samples, mixed standard solutions (100 μg/mL) were made by weighing and dissolving each reference standard in methanol.

### Metabolite profiling by UPLC/Q-TOF–MS

Metabolite profiling was performed on a UPLC system equipped with a high-pressure pump, automatic sample manager, and column heater (Waters Acquity, USA). A UPLC BEH C18 Column (2.1 mm × 50 mm, 1.7 μm) (Waters Acquity) and a C18 pre-column (2.1 mm × 5 mm, 1.7 μm) (Waters Acquity) were used. The mobile phase consisted of water with 0.1% (v/v) formic acid (A) and acetonitrile with 0.1% (v/v) formic acid (B). The gradient program was as follows: 0–1.5 min, 35–40% B; 1.5–7.0 min, 40–50% B; 7.0–10.0 min, 50–58% B; 10.0–11.8 min, 58–80% B; 11.8–12.3 min, 80–98% B; 12.3–14.0 min, 98% B; 14.0–14.5 min, 98–30% B; 14.5–16.0 min, 30% B. The injection volume was 1 µL. The target column temperature was 40 °C.

Chemical profile analysis was performed on a Xevo G2-S Q-Tof–MS analyzer (Waters Micromass, Manchester, UK) operated in both positive electrospray ionization modes. In the ESI^+^ mode, parameters were set as follows: mass range, 50–1500 Da; collision energy, 35–60 V; capillary voltage, 0.5 kV; cone voltage, 40 V; source temperature, 100 °C; desolvation gas temperature, 450 °C; cone gas flow rate, 50 L/h; desolvation flow rate, 900 L/h.

### Data pretreatment and statistical analyses

The centroid MS raw data were analyzed using the Progenesis QI 2.1 software (Waters, Milford, USA) for biomarker discovery. Multiple adduct ions, including [M + H]^+^, [M + Na]^+^, [2M + Na]^+^, [M + K]^+^, [M + H–H_2_O]^+^, and [M + NH4]^+^, were selected or self-edited to remove redundant adduct ion species. PCA and OPLS-DA were conducted in order to determine differences in the metabolic composition of normal and orange Danshen. The total ions of the samples were exported by Progenesis QI 2.1 software (Waters Co.).

### RNA sequencing, de novo transcriptome assembly, and analysis

Total RNA was extracted from the main root samples utilizing the Trizol method (Invitrogen, California, USA). RNA sequencing was performed on an Illumina HiSeq platform (Novogene Bioinformatics Technology Co. Ltd, China) for paired-end reads of 150–200 bp. Raw data reads were first processed to remove adaptors and low-quality bases in order to obtain clean reads for downstream analysis. Left- and right-sequenced reads were concatenated separately and submitted to Trinity to assemble a reference transcriptome with default parameters [15]. Clean reads were then sequenced and aligned to the reference transcriptome by Bowtie2 [16]; read counts for each gene were computed by in-house scripts. All de novo assemblages were searched against the NCBI Nr (E = 1.0E−5) and Nt (E = 1.0E−5), Swiss-Prot (E = 1.0E−5), and Pfam (E = 1.0E−2) databases. The KEGG pathway analysis was conducted using the KEGG automatic annotation server (E = 1.0E−10).

### Analysis of differentially expressed genes

The DEGs were analyzed using the DESeq 2 R package v1.16.1 [17], which provided statistical procedures for determining differential expression using a model based on the negative binomial distribution. The *p* values were adjusted using the Benjamini–Hochberg procedure to control for the false discovery rate. Genes with an adjusted *p* value < 0.05 were considered to be differentially expressed. The KOBAS software was used to test the statistical enrichment of DEGs in the KEGG pathways [18].

## Results

### PCA and OPLS-DA results

Lipophilic diterpenoid tanshinone compounds can be detected with high sensitivity in positive ion mode. Thus, UPLC/Q-TOF–MS in the positive electrospray ionization (ESI^+^) mode was employed to analyze the metabolites profiling of normal and orange Danshen. As can be seen in the total ion chromatogram, some peaks decreased or almost disappeared in the orange Danshen tanshinones (Fig. 1c). Progenesis QI was utilized for data pretreatment to generate a convenient dataset containing variables and observations.

**Fig. 2:**
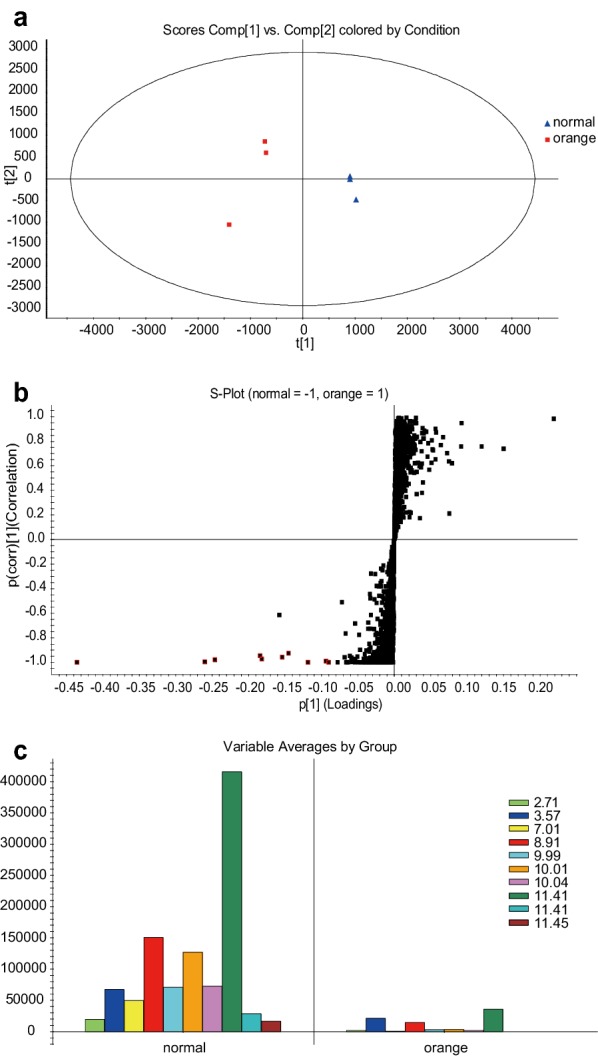
Chemometric analysis of normal and orange Danshen. **a** PCA analysis and score plot of normal and orange Danshen. **b** S-plot from the loading of the OPLS-DA. The x-axis indicates the variable contributions. The farther a data point lies from 0, the more it contributes to the variance of the sample. The y-axis indicates the samples’ correlations within the same sample group. The farther an exact mass/retention time pair lies from the value 0, the better its correlation with the injections. **c** Variable averages of ten biomarkers in normal Danshen identified by OPLS-DA

First, mass spectrometry (MS) data of samples in the ESI^+^ mode were statistically analyzed by untargeted principal component analysis (PCA), which was utilized to determine the similarity of the metabolite profiles among the fractions and retention time. Exact mass and ion intensity were used as variables. Points in the PCA score plot represent data observations; the closer the points, the more similar the data. Samples of normal and orange Danshen were well clustered and segregated into two different groups (Fig. 2a).

In order to determine the best discrimination of normal and orange Danshen and to discover the most important contributing variables, supervised orthogonal partial least-squares discriminant analysis (OPLS-DA) was employed for further analysis. A scatter plot (S-plot) was obtained from the loading of the OPLS-DA score plot, which highlighted the variables most responsible for differences among the groups (Fig. 2b). Each data point is an exact mass/retention time pair and corresponds to a marker ion. The x-axis showed the variable contributions. The further a data point was away from 0, the more it contributed to the variance. Based on S-plot from the OPLS-DA analysis (Fig. 2b), 10 potential markers (highlight in red in Fig. 2b) were detected and found to be higher in normal Danshen than in orange (Fig. 2c). It was indicates that the compound with a retention time of 11.41 min (i.e., tanshinone IIA) contributed to the most variation between the two groups.

### MS data analysis of the decreased tanshinones

Fragment ions of lipophilic diterpenoids were primarily derived from the losses of H_2_O (18 Da), CH_3_ (15 Da), and CO (28 Da). In total, 40 compounds were identified by comparing their exact molecular weight and MS fragmentation with the standard compounds or data found in published papers (Additional file 1: Table S1) [19–22]. Seven compounds were detected and found to decrease in orange Danshen. After comparison to the standard compounds, six were identified, including tanshindiol C (RT = 1.21 min), tanshindiol B (RT = 1.54 min), tanshinol B (RT = 2.71 min), tanshinone IIB (RT = 3.56 min), tanshinone (I RT = 8.94 min), and tanshinone IIA (RT = 11.44 min). Additionally, the compound with RT = 10.35 min was identified as 1,2-didehydrotanshinone IIA based on its exact molecular weight and MS fragmentation, based on the data reported by Yang et al. [20].

**Fig. 3:**
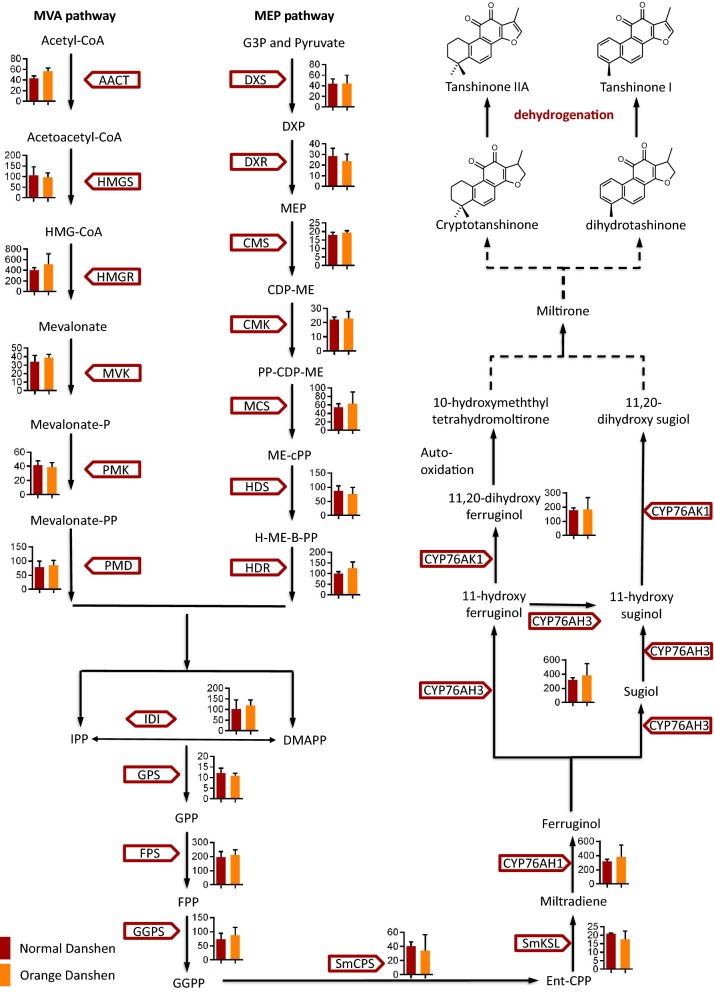
Expression of known functional genes involved in the biopathways of tanshinones. Isoprenoid pathway: MEP pathway: the 2-C-methyl-d-erythritol 4-phosphate pathway; MVA pathway: the mevalonate pathway. Intermediate metabolites in the pathway: Acetoacetyl-CoA: Acetoacetyl coenzyme A; Acetyl-CoA: Acetyl coenzyme A; CDP-ME: 4-(Cytidine 5′-diphospho)-2-C-methyl-d-erythritol; DMAPP: Dimethylallyl pyrophosphate; DXP: 1-Deoxy-d-xylulose 5-phosphate; Ent-CPP: Ent-copalyl diphosphate; FPP: Farnesyl diphosphate; G3P: Glyceraldehyde 3-phosphate; GGPP: Geranylgeranyl diphosphate; GPP: Geranyl diphosphate; H-ME-B-PP: 1-Hydroxy-2-methyl-2-butenyl 4-diphosphate; HMG-CoA: 3-Hydroxy-3-methylglutaryl CoA; IPP: Isopentenyl pyrophosphate; ME-cPP: 2-C-Methyl-d-erythritol 2,4-cyclodiphosphate; MEP: 2-C-Methyl-d-erythritol 4-phosphate; Mevalonate-P: Mevalonate-5-phosphate; Mevalonate-PP: Mevalonate-5-diphosphomevalonate; MVA: Mevalonate; PP-CDP-ME: 2-Phospho-4-(cytidine 5′-diphospho)-2-C-methyl-d-erythritol. Enzymes involving in the pathway: AACT: Acetoacetyl-CoA transferase; CMK: 4-(Cytidine 5′-diphospho)-2-C-methyl-d-erythritol kinase; CMS: 4-(Cytidine 5′-diphospho)-2-C-methyl-d-erythritol synthase; DXR: 1-deoxy-d-xylulose-5-phosphate reductoisomerase; DXS: 1-deoxy-d-xylulose-5-phosphate synthase; FPS: FPP synthase; GGPS: GGPP synthase; GPS: GPP synthase; HDR: 4-hydroxy-3-methyl-but-2-enyl diphosphate reductase; HDS: 4-Hydroxy-3-methylbut-2-enyl diphosphate synthase; HMGR: 3-Hydroxy-3-methylglutaryl CoA reductase; HMGS: Hydroxymethylglutaryl-CoA synthase; IDI: Isopentenyl-diphosphate delta-isomerase; MCS: 2-C-Methyl-d-erythritol 2,4-cyclodiphosphate synthase; MVK: Mevalonate kinase; PMD: Diphosphomevalonate decarboxylase; PMK: Phosphomevalonate kinase; SmCPS: *Salvia miltiorrhiza* copalyl diphosphate synthase; SmKSL: *Salvia miltiorrhiza* kaurene synthase-like

Although the confirmed compounds had distinct structures, they shared the same dehydrogenated furan ring. Specifically, tanshinone IIA and tanshinone I with long conjugated bonds were the terminal biosynthesis products of tanshinones and had a deep red color (Fig. 1b). The decrease in tanshinone IIA content appears to lead to root color change from red to orange. It is proposed that tanshinone IIA was converted from cryptotanshinone by dehydrogenation (Fig. 3). Meanwhile, tanshinone I was supposed to be converted from dihydrotanshinone I by dehydrogenation. This indicates that the reduction in tanshinone IIA, tanshinone I and so on may be due to the decreased expression of or degradation of enzymes that catalytically dehydrogenate substrates to products.

### Transcriptome profiling and expression of known functional genes in the biopathways of tanshinones

**Fig. 4:**
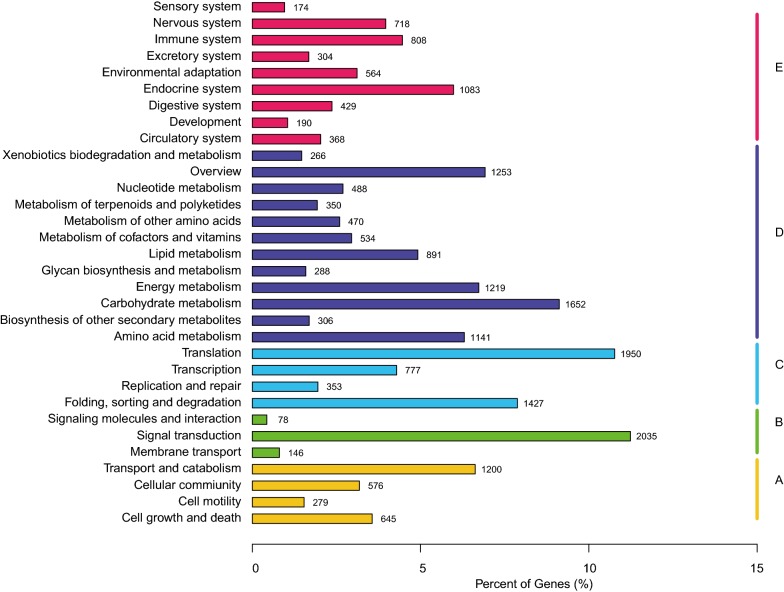
KEGG classification of the assembled unigenes. A Cellular processes; B environmental information processing; C genetic information processing; D metabolism; E organismal systems

In total, 189 million clean reads were generated for all samples, where 33,172,046, 31,992,708, and 29,460,962 were found in the three normal Danshen samples and 33,163,130, 31,364,922, and 30,122,148 were found in the three orange Danshen samples. The reference transcriptome assembled by using pooled reads contained 36,017 unigenes longer than 200 nt. Unigenes were searched against the NCBI Nr and Nt, KEGG, SwissProt, Pfam, and GO databases, and it was found that approximately 88.10% of the unigenes could be annotated in at least one of the databases. A total of 18,114 (~ 50%) unigenes could be annotated in KEGG, with 350 unigenes involved in the metabolism of terpenoids and polyketides, 108 involved in the biosynthesis of the terpenoid backbone, and 34 involved in diterpenoid biosynthesis (Fig. 4).

Tanshinones are abietane-type norditerpenoid quinone natural products. Diterpenoids have been reported to originate in the 2-C-methyl-d-erythritol 4-phosphate pathway (MEP pathway) of plastids, however, cross-talk does occur between the mevalonate pathway (MVA pathway) and MEP pathways during the biosynthesis of tanshinones [23, 24]. Genes involved in the biosynthesis of the terpenoid backbone [i.e., isopentenyl pyrophosphate (IPP) and dimethylallyl pyrophosphate (DMAPP)] have been investigated in normal and orange Danshen. From the common diterpenoid precursor (i.e., geranylgeranyl diphosphate (GGPP)) to the metabolic intermediates of tanshinones, five genes are responsible for tanshinone biosynthesis and have been characterized, including two diterpenoid synthases (i.e., SmCPS1 and SmKSL1) [25, 26], as well as three cytochrome P450 s (i.e., CYP76AH1, CYP76AH3, and CYP76AK1) [27, 28]. In this study, the expression of upstream genes in normal and orange Danshen did not differ significantly (Fig. 3). Additionally, in the metabolic profiling there was no significant difference observed in the biopathway intermediates, including cryptotanshinone, dihydrotanshinone, and the intermediate sugiol. This indicates that there is a balanced metabolic flow upstream.

### KEGG enrichment analysis of down-regulated genes

**Fig. 5:**
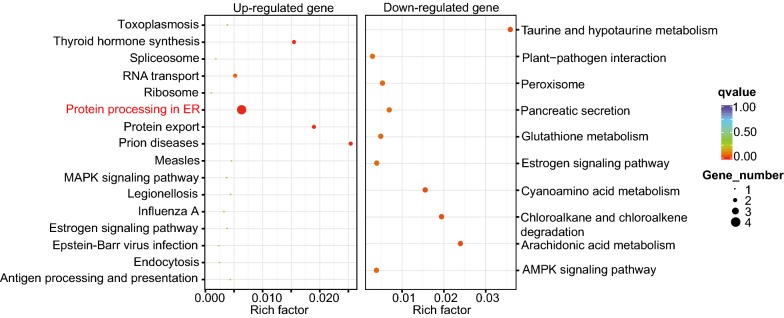
Scatterplot of the KEGG pathway of significantly enriched DEGs in orange Danshen. The rich factor refers to the ratio of the number of DEGs to the total number of genes enriched in a specific category

In total, 104 genes were significantly expressed in normal and orange Danshen (with adjust *p* value < 0.05; Additional file 2: Table S2, Additional file 3). In order to understand which categories are overrepresented, differentially expressed genes (DEGs) were further analyzed by a KEGG enrichment analysis. In total, there were 58 down-regulated genes in orange Danshen. The KEGG pathway analysis mapped 10 categories (Fig. 5), including cyanoamino acid metabolism, peroxisome, the AMP-activated protein kinase (AMPK) signaling pathway, and plant-pathogen interactions (Additional file 4: Table S3). The annotated function of these genes, based on other databases, included glutamyltranspeptidase, glutathione hydrolase, peptidylprolyl isomerase, hydroxy fatty acid phosphatase, and epoxide hydrolase activities (Additional file 2: Table S2).

Metabolic profiling revealed that compounds with C_15_-C_16_ double bonds in the furan ring were the principal structures found in normal and orange Danshen. Most dehydrogenation reactions were catalyzed by cytochrome P450s, NADP/NAD-dependent dehydrogenases, and reductases, thus, we selected these gene families for further analysis. Additionally, down-regulated genes related to reductase or dehydrogenase catalytic activity that were highly expressed in normal Danshen were also selected. However, there were no genes related to dehydrogenation with expression profiles concordant with the accumulation of tanshinone IIA or tanshinone I. Therefore, genes that were up-regulated in orange Danshen were the focus.

### KEGG enrichment analysis of up-regulated genes

There were 46 genes that were up-regulated in orange Danshen based on the DESeq analysis. In total, 16 significant KEGG categories were identified, including protein processing in the endoplasmic reticulum (ER), RNA transport, and protein export (Fig. 5 and Table S3). Interestingly, four genes were assigned to the “protein processing in the ER.” In the up-regulated genes, these four genes were annotated as BIPs (i.e., c80749_g1, up-regulated by 5.2 fold; and c80749_g2, up-regulated by 5.9 fold), heat shock protein 70 kDa (i.e., c91931_g1, whose read count value was up-regulated from 0 to 112.5), and a molecular chaperone (i.e., c75193_g1, up-regulated by 2.6 fold).

In the ER, correctly folded proteins are packaged into transport vesicles, while incorrectly folded proteins are retained in the complex with molecular chaperones and bind to BIP, then are directed towards degradation, which is mediated by heat shock proteins and molecular chaperones [29]. The four genes related to ER-associated protein degradation were up-regulated in orange Danshen, while there was no identifiable down-regulated gene responsible for catalytic C_15_-C_16_ dehydrogenation, which indicates that there is an alternative mechanism related to the decrease of tanshinone IIA content. Based on the transcriptome analysis, it appears that the low accumulation of tanshinone IIA and related tanshinones with dehydrogenated furan rings may be a result of the degradation of corresponding enzymes that may catalyze C_15_–C_16_ dehydrogenase, rather than the lower expression of relative genes.

In addition to the genes assigned to protein processing in the ER, there was one gene annotated as a zinc-finger protein and two genes annotated as eukaryotic translation initiation factor 5B (*eIF5B*) were up-regulated. Zinc-finger proteins and *eIF5B* have been previously reported to play a role in stress resistance [30–32]. Up-regulation of zinc-fingers and eIF5B indicates that orange Danshen may have experienced stress during cultivation.

## Discussion

Accumulation of tanshinones results in a red phenotype in Danshen. In this study, during resource investigation, samples with orange roots were found to occur in a different cultivation base, suggesting that the concentration or constituents of tanshinones in orange Danshen may differ from normal Danshen. UPLC/Q-TOF–MS analysis revealed that the peaks of tanshinone IIA and tanshinone I almost disappeared in orange Danshen. In total, seven tanshinones with dehydrogenated furan rings were decreased. Tanshinone IIA with long conjugated bonds had a deep red color, accounting for 25.3–63.4% of the total tanshinones and are the terminal products of tanshinone biosynthesis [19]. Low concentration of tanshinone IIA and related tanshinones, such as tanshinone I, with a double C_15_-C_16_ bond led to an orange phenotype in cultivated fields.

Tanshinone IIA, cryptotashinone, tanshinone I, and dihydrotanshinone I are index components of Danshen [33]. Specifically, tanshinone IIA is an indicative lipophilic ingredient in the Pharmacopoeia of the People’s Republic of China [1]. Sodium sulfonate forms of tanshinone IIA have been widely used to treat coronary heart disease, angina pectoris, and myocardial infarction in the clinical setting [34]. Thus, the decreased content of tanshinone IIA and other related compounds would detrimentally affect the quality of Danshen and its utilization.

Decreased expression or degradation of the enzymes involved in catalytic dehydrogenation may result in low transformation efficiency of tanshinones I and IIA from dihydrotanshinone I and cryptotanshinone, respectively. However, there was not a gene detected, which was responsible for catalytic dehydrogenation in the down-regulated genes of orange Danshen based on the comparative transcriptome analysis. However, two genes have been reported to be related to stress [30–32, 35], and four genes related to ER-associated protein degradation [29], which were found to be significantly up-regulated in orange-Danshen.

Zinc-finger proteins have been reported to play a central role in abiotic stress resistance, especially oxidative stress in Arabidopsis, during which the expression of both zinc-finger and heat-shock proteins increased [30, 31]. The *eIF5B* mutant of Arabidopsis behaves as the wild type in the absence of stress, although it is more temperature sensitive, which indicates that eIF5B plays a role in stress resistance [32]. Moreover, it interacts with other initiation factors and GTP to initiate translation, and has been reported to have the ability to prevent thermal aggregation and promote refolding of heat-labile proteins, such as chaperone-like activity in *Pisum sativum* [35]. It was proposed that Danshen cultivated in field would suffer from various pathogens, and epidemiology [36]. Danshen with orange roots might be suffered from stress or disease. This resulted in unusual initiation of genes responsible for catalytic dehydrogenation, which might further result in uncorrected folding of the corresponding proteins. The uncorrected folding proteins were then guided to ER-associated protein degradation, and finally interrupted the dehydrogenation step in biopathway of tanshinones.

## Conclusions

In this study, metabolome and transcriptome analyses were integrated to analyze the orange Danshen from cultivated fields. Metabolome analyses indicated that low concentration of tanshinone IIA and related tanshinones, such as tanshinone I, with a double C_15_–C_16_ bond resulted an orange phenotype of Danshen in cultivated fields. During cultivation, both biotic and abiotic stressors are actively present throughout the growth period of Danshen. Up-regulated zinc-finger proteins and eIF5s indicate stress resistance during cultivation. Danshen roots that experience stress may result in unusual transcription initiation and incorrect protein folding, which may lead to the degradation of proteins related to the dehydrogenation of tanshinones in C_15_–C_16_ bonds. This limited dehydrogenation of cryptotanshinone and dihydrotanshinone I into tanshinones IIA and I products, respectively, leads to a reduced quality of Danshen in cultivated fields. Future studies should functionally characterize the exact genes involved in catalytic dehydrogenation of furan rings, and investigate the degradation mechanism and incorrect folding of catalytic enzymes that depend on zinc-finger proteins, heat-shock proteins, and eIF. In conclusion, because quality degradation often takes place during cultivation [36], the data presented here and the proposed mechanism in this study will provide a reference for the cultivation of Chinese material medicine.

## Supplementary information

**Additional file 1: Table S1.** Identification of tanshinones by UPLC/Q-TOF–MS in the ESI^+^ mode.

**Additional file 2: Table S2.** Gene annotation of DEGs in normal and orange Danshen with *pad*j < 0.05.

**Additional file 3.** Gene sequences of 104 significantly differentially expressed genes in normal and orange Danshen (with *padj* < 0.05).

**Additional file 4: Table S3.** KEGG pathway enrichment analysis of DEGs in normal and orange Danshen with *padj *< 0.05.

## Data Availability

The datasets supporting the conclusions of this article are included within the article and its additional files.

## Abbreviations

UPLC/Q-Tof-MS: ultra-high-performance liquid chromatography-quadrupole time-of-flight mass spectrometry
KEGG: Kyoto Encyclopedia of Genes and Genomes
ER: endoplasmic reticulum
MS: mass spectrometry
ESI: electrospray ionization
DEG: differentially expressed genes
PCA: principal component analysis
OPLS-DA: orthogonal partial least-squares discriminant snalysis
MEP pathway: the 2-C-methyl-d-erythritol 4-phosphate pathway
MVA pathway: the mevalonate pathway
Acetoacetyl-CoA: acetoacetyl coenzyme A
Acetyl-CoA: acetyl coenzyme A
CDP-ME: 4-(cytidine 5′-diphospho)-2-C-methyl-d-erythritol
DMAPP: dimethylallyl pyrophosphate
DXP: 1-deoxy-d-xylulose 5-phosphate
Ent-CPP: ent-copalyl diphosphate
FPP: farnesyl diphosphate
G3P: glyceraldehyde 3-phosphate
GGPP: geranylgeranyl diphosphate
GPP: geranyl diphosphate
H-ME-B-PP: 1-hydroxy-2-methyl-2-butenyl 4-diphosphate
HMG-CoA: 3-hydroxy-3-methylglutaryl CoA
IPP: isopentenyl pyrophosphate
ME-cPP: 2-C-methyl-d-erythritol 2,4-cyclodiphosphate
MEP: 2-C-methyl-d-erythritol 4-phosphate
Mevalonate-P: mevalonate-5-phosphate
Mevalonate-PP: mevalonate-5-diphosphomevalonate
MVA: mevalonate
PP-CDP-ME: 2-phospho-4-(cytidine 5′-diphospho)-2-C-methyl-d-erythritol
AACT: acetoacetyl-CoA transferase
CMK: 4-(cytidine 5′-diphospho)-2-C-methyl-d-erythritol kinase
CMS: 4-(cytidine 5′-diphospho)-2-C-methyl-d-erythritol synthase
DXR: 1-deoxy-d-xylulose-5-phosphate reductoisomerase
DXS: 1-deoxy-d-xylulose-5-phosphate synthase
FPS: FPP synthase
GGPS: GGPP synthase
GPS: GPP synthase
HDS: 4-hydroxy-3-methylbut-2-enyl diphosphate synthase
HMGR: 3-hydroxy-3-methylglutaryl CoA reductase
HMGS: hydroxymethylglutaryl-CoA synthase
IDI: Isopentenyl-diphosphate delta-isomerase
MCS: 2-C-methyl-d-erythritol 2,4-cyclodiphosphate synthase
MVK: mevalonate kinase
PMD: diphosphomevalonate decarboxylase
PMK: phosphomevalonate kinase
SmCPS: *Salvia miltiorrhiza* copalyl diphosphate synthase
SmKSL: *Salvia miltiorrhiza* kaurene synthase-like
