# Supplementary material for: Metabolome and transcriptome analyses reveal quality change in the orange-rooted Salvia miltiorrhiza (Danshen) from cultivated field
Source: Chin Med. 2019 Oct 2;14:42. doi: 10.1186/s13020-019-0265-6 (PMC6775661; doi:10.1186/s13020-019-0265-6)
Supplement: Supplementary file 1 — Additional file 1: Table S1. Identification of tanshinones by UPLC/Q-TOF–MS in the ESI+ mode. [file 13020_2019_265_MOESM1_ESM.docx]

Additional table 1. Identification of tanshinones from extraction of Danshen in positive mode ([M+H]^+^) by UPLC/Q-TOF-MS.

| Peak  (No.) | RT  (min) | Measured  Mass(m/z) | Calculated  Mass(m/z) | Error  (ppm) | Formula | Identification | Variation ^a^ | MS/MS(m/z) | Reference |
| --- | --- | --- | --- | --- | --- | --- | --- | --- | --- |
| 1 | 0.82 | 315.1215 | 315.1232 | -5.4 | C_18_H_18_O_5_ | 15,16-Dihydrotanshindiol B |  | 521.1052,315.1215,295.0608,277.0473,249.0544,221.0592,181.0508,165.0696,139.0382 | [[1](#_ENREF_1)] |
| 2 | 0.94 | 315.1215 | 315.1232 | -5.4 | C_18_H_18_O_5_ | 15,16-Dihydrotanshindiol C |  | 315.1215,295.0608,277.0473,249.0512,181.0505,150.1274,139.0406 | [[1](#_ENREF_1)] |
| 3 | 0.99 | 313.1056 | 313.1076 | -6.4 | C_18_H_16_O_5_ | Tanshindiol A |  | 647.0671,355.0831,295.0573,249.0544,221.0562,165.0723,150.1274,136.1110 |  |
| 4 | 1.21 | 313.1093 | 313.1076 | 5.4 | C_18_H_16_O_5_ | Tanshindiol C |  | 647.1816,335.0887,313.1093,295.0959,267.1003,249.1545,227.1749,164.1431,150.1274,136.1110,122.0977 |  |
| 5 | 1.54 | 313.1093 | 313.1076 | 5.4 | C_18_H_16_O_5_ | Tanshindiol B |  | 335.0925,313.1093,297.1095,267.1037,249.1578,227.1749,164.1458,150.1274,136.1133,122.0954 |  |
| 6 | 1.74 | 313.1454 | 313.1440 | 4.5 | C_19_H_20_O_4_ | Miltionone Ⅱ |  | 313.1454,290.2663,269.1537,238.0663,223.0406,178.0820,165.0696,153.0718,141.0694,128.0618,115.0548 | [[2](#_ENREF_2)] |
| 7 | 2.19 | 283.0966 | 283.0970 | -1.4 | C_17_H_14_O_4_ | Phenanthro[1,2-b]furan-6,10,11(7H)-trione,1,2,8,9-tetrahydro-1-methyl-, (R)- |  | 283.0966,227.1749,189.0734,165.0696,150.1274,136.1133 | [[3](#_ENREF_3)] |
| 8 | 2.62 | 295.0959 | 295.0970 | -3.7 | C_18_H_14_O_4_ | Trijuganone A |  | 317.0782,295.0959,214.0921,178.0766,150.1274,136.1110,122.0954 | [[3](#_ENREF_3)] |
| 9 | 2.71 | 297.1130 | 297.1127 | 1.0 | C_18_H_16_O_4_ | Tanshinol B |  | 615.1961,319.0958,297.1130,279.1010,261.0898,241.1664,219.1865,150.1274,136.1110,122.0977 |  |
| 10 | 2.93 | 311.1287 | 311.1283 | 1.3 | C_19_H_18_O_4_ | 3alpha-Hydroxytanshinone IIA |  | 333.1144,311.1287,279.1010,249.1578,165.0775,141.0742,135.1080 | [[2](#_ENREF_2)] |
| 11 | 3.21 | 295.0959 | 295.0970 | -3.7 | C_18_H_14_O_4_ | 3-Hydroxymethylenetanshinquinone |  | 317.0782,295.0959,227.1779,214.0891,205.0688,178.0793,150.1274,136.1133 | [[2](#_ENREF_2)] |
| 12 | 3.56 | 311.1287 | 311.1283 | 1.3 | C_19_H_18_O_4_ | Tanshinone IIB |  | 643.2325,333.1107,311.1287,293.1173,274.2758,251.1055,230.2500,214.0921,164.1431,150.1274,136.1133,122.0977 |  |
| 13 | 3.99 | 341.1373 | 341.1389 | -4.7 | C_20_H_20_O_5_ | Cryptomethyltanshinoate |  | 341.1373,249.1513,227.1779,214.0891,191.0872,165.0696,150.1274,136.1133 | [[2](#_ENREF_2)] |
| 14 | 4.31 | 341.1373 | 341.1389 | -4.7 | C_20_H_20_O_5_ | Trijuganone C |  | 363.1191,341.1373,288.2901,263.1068,235.1116,202.0771,192.0924,178.0793,165.0696,150.1274,136.1110,122.0977 | [[4](#_ENREF_4)] |
| 15 | 4.42 | 327.1266 | 327.1232 | 10.4 | C_19_H_18_O_5_ | 3-Hydroxtanshinone IIB |  | 327.1266,281.1553,227.1779,178.0793,165.0696,150.1274,141.0645,136.1110 | [[3](#_ENREF_3)] |
| 16 | 4.45 | 299.1652 | 299.1647 | 8.5 | C_19_H_22_O_3_ | Miltiodiol |  | 321.1455,299.1652,281.1518,191.0844,178.0766,165.0749,150.1274,136.1110 | [[2](#_ENREF_2)] |
| 17 | 4.62 | 297.1130 | 297.1127 | 1.0 | C_18_H_16_O_4_ | Tanshinone VI |  | 297.1130,279.1010,249.1545,189.0678,165.0670,150.1274,136.1133 | [[1](#_ENREF_1)] |
| 18 | 4.67 | 309.1149 | 309.1127 | 7.1 | C_19_H_16_O_4_ | Tanshinaldehyde |  | 309.1149,297.1059,287.2005,279.1010,189.0678,165.0723,150.1274,136.1110 | [[3](#_ENREF_3)] |
| 19 | 5.31 | 297.1130 | 297.1127 | 1.0 | C_18_H_16_O_4_ | Danshenxinkun A |  | 297.1130,279.1010,253.1616,227.1718,165.0723,150.1274,136.1133 | [[1](#_ENREF_1)] |
| 20 | 5.49 | 299.1652 | 299.1647 | 1.7 | C_19_H_22_O_3_ | Sageone |  | 619.3129,321.1491,299.1652,281.1553,227.1749,150.1274,136.1133 |  |
| 21 | 5.89 | 279.1044 | 279.1021 | 8.2 | C_18_H_14_O_3_ | Dihydrotanshinone I |  | 579.1814,301.0861,279.1044,261.0931,251.1087,233.0991,205.1040,189.0706,164.1431,150.1274,136.1133,122.0954 |  |
| 22 | 5.89 | 279.1044 | 279.1021 | 8.2 | C_18_H_14_O_3_ | Dihydroisotanshinone I |  | 579.1814,301.0861,279.1044,261.0931,251.1087,233.0991,205.1040,189.0706,164.1431,150.1274,136.1133,122.0954 |  |
| 23 | 5.89 | 279.1044 | 279.1021 | 8.2 | C_18_H_14_O_3_ | Methylenetanshinquinone |  | 579.1765,301.0861,279.1044,205.1010,189.0706,178.0793,165.0723,150.1274,141.0694,136.1133,115.0548 |  |
| 24 | 6.58 | 281.1205 | 281.1178 | 9.6 | C_18_H_16_O_3_ | Danshenxinkun B |  | 583.2057,303.0985,281.1175,263.1068,235.1116,219.0806,202.0771,191.0844,178.0793,165.0696,152.0636,141.0694,115.0548 |  |
| 25 | 6.59 | 281.1175 | 281.1178 | -1.1 | C_18_H_16_O_3_ | Methylene dihydrotanshinone |  | 583.2106,303.0985,281.1175,263.1068,235.1116,202.0771,191.0844,178.0793，165.0723,150.1274,136.1133 | [[2](#_ENREF_2)] |
| 26 | 6.60 | 281.1175 | 281.1178 | -1.1 | C_18_H_16_O_3_ | Tetrahydro tanshinone I |  | 583.2106,303.0985,281.1175,263.1068,235.1116,202.0771,191.0844,178.0793,165.0723,150.1274,136.1133 |  |
| 27 | 6.86 | 313.1454 | 313.1440 | 4.5 | C_19_H_20_O_4_ | Miltionone I |  | 313.1454,178.0820,165.0723,150.1274,141.0694,136.1133 | [[2](#_ENREF_2)] |
| 28 | 6.92 | 315.1578 | 315.1596 | -5.7 | C_19_H_22_O_4_ | Neocryptotanshinone |  | 315.1578,299.2006,189.0706,178.0766,165.0670,150.1274,136.1110,115.0548 | [[4](#_ENREF_4)] |
| 29 | 6.99 | 339.1226 | 339.1232 | -1.8 | C_20_H_18_O_5_ | Methyltanshinonate |  | 699.2145,361.1027,339.1226,315.1578,299.2041,279.1010,261.0898,233.0959,205.1010,189.0706,150.1274,136.1110 |  |
| 30 | 7.01 | 279.1010 | 279.1021 | -3.9 | C_18_H_14_O_3_ | 1,2-Dihydrotanshinquinone |  | 279.1010,261.0898,233.0991,205.1010,165.0723,150.0724,136.1110 |  |
| 31 | 8.51 | 297.1483 | 297.1491 | -2.7 | C_19_H_20_O_3_ | Isocryptotanshinone |  | 615.2722,319.1287,297.1483,282.1234,279.1385,268.1076,254.0934,251.1444,237.0856,221.0987,189.0678,178.0793,165.0696,150.1274 |  |
| 32 | 8.51 | 297.1483 | 297.1491 | -2.7 | C_19_H_20_O_3_ | Cryptotanshinone |  | 615.2722,319.1287,297.1483,282.1234,279.1385,268.1076,254.0934,251.1444,237.0856,221.0987,189.0678,178.0793,165.0696,150.1274 |  |
| 33 | 8.94 | 277.0882 | 277.0865 | 6.1 | C_18_H_12_O_3_ | Tanshinone I |  | 575.1465,299.0697,277.0882,249.0932,178.0793,164.1431,150.1274,136.1133,122.0977 |  |
| 34 | 9.28 | 301.2174 | 301.2168 | 2.0 | C_20_H_28_O_2_ | Sugiol |  | 301.2174,213.1263,198.1041,171.0817,163.0808,150.1274,136.1133 |  |
| 35 | 9.64 | 265.1248 | 265.1229 | 7.2 | C_18_H_16_O_2_ | Miltirone I |  | 551.1289,287.1069,265.1248,236.1196,203.0816,178.0793,165.0749,150.1299,136.1133 | [[2](#_ENREF_2)] |
| 36 | 10.35 | 293.1173 | 293.1178 | -1.7 | C_19_H_16_O_3_ | 1,2-Didehydrotanshinone IIA |  | 315.0997,293.1173,179.0881,165.0723,150.1299,136.1133 | [[2](#_ENREF_2)] |
| 37 | 10.61 | 287.1658 | 287.1647 | 3.8 | C_18_H_22_O_3_ | Cryptoacetalide/ Epi-Cryptoacetalide |  | 287.1658,267.1438,178.0793,153.0718,150.1274,136.1133,91.0558 | [[2](#_ENREF_2)] |
| 38 | 11.12 | 281.1553 | 281.1542 | 3.9 | C_19_H_20_O_2_ | Dehydromiltirone |  | 303.1377,281.1553,205.1010,178.0766,165.0723,150.1274,136.1133 |  |
| 39 | 11.44 | 295.1346 | 295.1334 | 4.1 | C_19_H_18_O_3_ | Tanshinone IIA |  | 611.2397,317.1183,295.1346,277.1223,249.1287,235.0771,219.0806,189.0706,178.0793,165.0723,150.1299,136.1133,122.0977 |  |
| 40 | 11.87 | 283.1724 | 283.1698 | 9.2 | C_19_H_22_O_2_ | Miltirone |  | 587.3152,305.1534,283.1724,268.1478,265.1614,240.1160,223.1139,189.0706,178.0793,165.0723,152.0636,150.1274,136.1133,122.0977 |  |

^a^ Arrow indicated that content of the compound was decreased on orange Danshen.

**References**

1. Yang M, Liu A, Guan S, Sun J, Xu M, Guo D: Characterization of tanshinones in the roots of *Salvia miltiorrhiza* (Dan-shen) by high-performance liquid chromatography with electrospray ionization tandem mass spectrometry. Rapid Commun Mass Spectrom. 2006;20(8):1266-80.

2. Xie W, Zhang H, Zeng J, Chen H, Zhao Z, Liang Z: Tissues-based chemical profiling and semi-quantitative analysis of bioactive components in the root of *Salvia miltiorrhiza* Bunge by using laser microdissection system combined with UPLC-q-TOF-MS. Chem Cent J. 2016;10(42):1-13.

3. Yang ST, Wu X, Rui W, Guo J, Feng YF: UPLC/Q-TOF-MS analysis for identification of hydrophilic phenolics and lipophilic diterpenoids from Radix *Salviae Miltiorrhizae*. Acta Chromatographica. 2015;27(4):711-28.

4. Zhao Q, Song Z, Fang X, Pan Y, Guo L, Liu T, Wang J: Effect of genotype and environment on *Salvia miltiorrhiza* roots using LC/MS-based metabolomics. Molecules. 2016;21(4):1-17.
